# The measles emergency is over, but the crisis continues – a call to action for the Pacific Islands

**DOI:** 10.7189/jogh.10.020301

**Published:** 2020-12

**Authors:** Annette Kaspar, Sione Pifeleti, Bernard CS Whitfield

**Affiliations:** 1 Hearing Research Unit for Children, School of Health and Rehabilitation Sciences, University of Queensland, Brisbane, Australia; 2 ENT Clinic, Tupua Tamasese Meaole Hospital, Apia, Samoa; 3 Australian Volunteer Program (AVP), Australia; 4 ENT Department, Logan Hospital, Queensland, Australia; 5 Griffith University, School of Medicine, Queensland, Australia

On 15 November 2019, the Government of Samoa declared a state of emergency for the country due to a measles epidemic. The following six weeks were marked by closure of schools, prohibition of children at public gatherings, intensive immunization campaigns, curfews, and re-distribution of health resources and services to manage the overwhelming influx of measles patients. The state of emergency was lifted on the 29th of December 2019. According to the Joint WHO/UNICEF Pacific Islands Measles Outbreak Situation Report (8 January 2020), there was a total of 5697 measles cases (Total population of Samoa in 2016: 195 000) [1]. There were 83 measles-related deaths, with 87% of these among children under 5 years of age. There were 1860 measles-related hospital admissions, and 95% of cases recovered and were discharged.

Samoa is now in a state of recovery. Although the measles emergency is over, the health system should now turn its attention to managing the complications that may arise following acute measles infection. This includes the permanent disabilities of hearing, visual, and cognitive impairment [2-4]. It is also a time to review the national immunization program, and investigate areas for improvement.

Research audiologist (author AK) is on assignment in Samoa for two years, and has previously published a viewpoint in this journal advocating for a public health approach to childhood hearing impairment in the Pacific Islands [5]. The current state of recovery in Samoa presents an ideal opportunity for the Ear, Nose, and Throat (ENT) Clinic to implement this principle. The Acting Head of the ENT Clinic and Surgeon (author SP) was a member of the Ministry of Health planning committees during the measles crisis, and continues to be part of the recovery committee. He is ideally placed to advocate for the inclusion of surveillance/screening for ear and hearing disorders among measles survivors in the recovery plan.

Measles outbreaks were also reported in Fiji, Tonga, and American Samoa during October-December 2019. The aim of this viewpoint is to highlight the Samoan experience, and to urge other Pacific Islands nations to review their advocacy, approach, and management for preventable disease and disability. The Sustainable Development Goal (SDG) Project offers an ideal platform to implement strategies aimed at improving immunization programs, as well as improving childhood developmental health through public health service delivery.

## STATE OF RECOVERY FOLLOWING THE MEASLES EMERGENCY: AN OPPORTUNITY TO ADVOCATE FOR TARGETED SURVEILLANCE/SCREENING OF COMPLICATIONS

According to the WHO Global Health Observatory, the first-dose measles immunization coverage among 1-year-old Samoan children in 2018 was only 31%, well below the recommended level of 95% (**Table 1**) [6]. This number decreased further when the National Immunization Program was suspended from July 2018 to April 2019, and there was a recall of all Measles/Mumps/Rubella vaccines following the deaths of two small children. The decline in herd immunity, the global spread of the measles virus, and the high-volume of travel between Samoa and neighbouring measles-affected countries were all contributing factors that ultimately led to the measles outbreak in Samoa [7].

**Table 1:** Overview of measles immunization coverage in the Pacific Islands in 2018*

Country	Measles-containing-vaccine first-dose (MCV1) immunization coverage among 1-y-olds (%)	Measles-containing-vaccine second-dose (MCV2) immunization coverage by the nationally recommended age (%)
Cook Islands	99	99
Fiji	94	94
Federated States of Micronesia	73	48
Kiribati	84	79
Nauru	99	94
Niue	99	99
Republic of the Marshall Islands	83	61
Samoa	31	13
Solomon Islands	93	54
Tonga	85	85
Tokelau	Not available	Not available
Tuvalu	88	81
Vanuatu	75	Not available

*Source: WHO Global Health Observatory Data Repository [6].

Following the intensive measles immunization campaign of November-December 2019, the Government of Samoa reported achieving the desired immunization coverage rate for the country. One of the aims of the current state of recovery is to ensure that the recommended immunization coverage rates for both first and second doses of measles-containing vaccines is achieved and maintained. The table below summarizes the measles immunization status from other Pacific Island nations, and efforts are currently under way to ensure all those susceptible, especially 6 to 59 month-old children, are vaccinated as recommended [1].

Measles infection is a risk-factor for permanent hearing, visual, and cognitive impairments [3,4,8]. At this time, there is little routine surveillance/screening among children in the Pacific Islands who are at-risk of these neurological complications following causal infections such as measles. The current state of recovery presents an opportunity to actively review children who survived the recent measles epidemic for any permanent sensory impairments. These activities may provide a foundation for routine targeted surveillance/screening, not only for measles, but other infectious causes of permanent disability (eg, meningitis).

**Figure:**
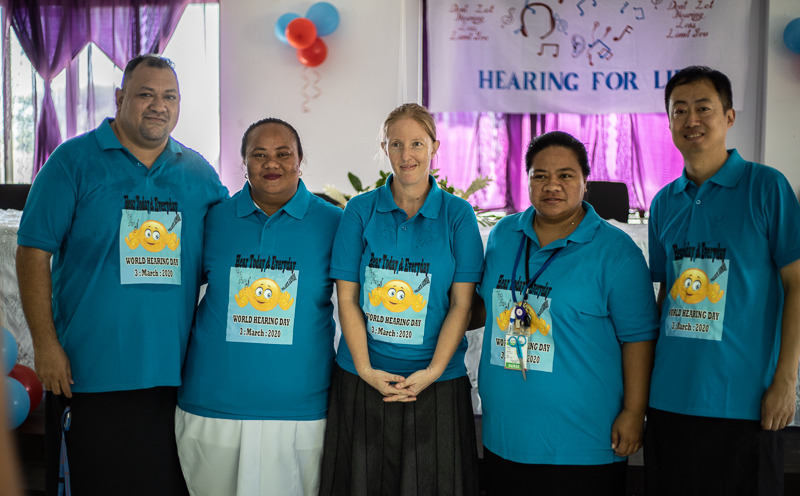
Photo: ENT Team of Samoa on World Hearing Day 2020 (from the team’s collection, used with permission).

The ENT Clinic is collaborating with the recovery effort to review all measles survivors for ear and hearing disorders. The ENT Clinic will conduct a study on the prevalence and pattern of ear disease and hearing loss among measles survivors in Samoa in order to highlight this health issue, and provide support for on-going and future prevention activities. Otitis media (OM) is a common ear disease that is often present secondary to measles infection [2]. Given that OM is already a significant public health problem among Pacific Island children, review by the ENT Clinic is essential to ensure optimal diagnosis and treatment [9]. OM that is poorly managed may lead to chronic and potentially life-threatening complications [9].

All measles survivors who present to the ENT Clinic will also receive a hearing assessment by the newly established Audiology Clinic. Children with OM usually present with a temporary hearing loss that will often resolve upon successful management of the ear disease. In addition to OM-related hearing loss, the measles infection itself may also cause permanent damage to the cochlear (ie, inner ear), and the resulting hearing loss is irreversible and often profound for both ears [8]. Early identification and intervention for children with a permanent hearing disability is critical for obtaining optimal language and development outcomes. In Samoa, the SENESE Inclusion Education Support Services are able to provide intervention options for hearing impairment, including provision of amplification devices (ie, hearing aids) and instruction in Sign Language.

The ENT Clinic used World Hearing Day 2020 (3rd March) to highlight measles as a preventable cause of permanent hearing loss. As well as providing key ear health messages aimed at reducing ear disease and associated hearing loss, this was an opportunity to promote the role of childhood immunizations for protection against childhood mortality, as well as irreversible conditions such as permanent deafness.

The ENT Clinic would encourage specialist colleagues to similarly engage and develop a targeted surveillance/screening program for other preventable neurological disabilities. The Ophthalmology Clinic is advocating eye and vision assessments to identify any cases of keratoconjunctivitis, a major complication of measles that can lead to blindness. The current goodwill shown to Samoa to provide assistance in the post-measles recovery period may be maximised to implement long-term public health surveillance/screening programs, especially for children. These aims would contribute to achieving SDGs in Samoa regarding child health, education, and disability.

## CONCLUSION

The state of recovery in Samoa is an ideal opportunity to review immunization programs and other health promotion messages. The risk of permanent sensory disability among measles survivors should be addressed during recovery efforts through surveillance/screening programs for any ear/hearing and eye/vision disorders. The tragedy of the measles emergency in Samoa may serve as a catalyst to improve immunization coverage rates and reduce preventable childhood disabilities across the Pacific Islands.
